# Designing development programs for non-traditional antibacterial agents

**DOI:** 10.1038/s41467-019-11303-9

**Published:** 2019-07-31

**Authors:** John H. Rex, Holly Fernandez Lynch, I. Glenn Cohen, Jonathan J. Darrow, Kevin Outterson

**Affiliations:** 1 F2G Limited, Eccles, Cheshire, M30 0LX UK; 2 McGovern Medical School at The University of Texas Health Science Center at Houston, Houston, TX 77030 USA; 3 University of Pennsylvania Perelman School of Medicine, Philadelphia, PA 19104 USA; 4 Harvard Law School, Cambridge, MA 02138 USA; 5 Petrie-Flom Center, Cambridge, MA 02138 USA; 6 Harvard Medical School, Boston, MA 02115 USA; 7 Boston University School of Law, CARB-X, Boston, MA 02215 USA

**Keywords:** Antimicrobials, Clinical trial design, Drug discovery

## Abstract

In the face of rising rates of antibacterial resistance, many responses are being pursued in parallel, including ‘non-traditional’ antibacterial agents (agents that are not small-molecule drugs and/or do not act by directly targeting bacterial components necessary for bacterial growth). In this Perspective, we argue that the distinction between traditional and non-traditional agents has only limited relevance for regulatory purposes. Rather, most agents in both categories can and should be developed using standard measures of clinical efficacy demonstrated with non-inferiority or superiority trial designs according to existing regulatory frameworks. There may, however, be products with non-traditional goals focused on population-level benefits that would benefit from extension of current paradigms. Discussion of such potential paradigms should be undertaken by the development community.

## Abstract (web summary)

Non-traditional antibacterial agents are challenging to develop. In this Perspective, the authors argue that the distinction between traditional and non-traditional agents has only limited relevance for regulatory purposes, although products with non-traditional goals focused on population-level benefits might benefit from extension of current paradigms.

## Introduction

Given the threats posed by the rise of antibacterial resistance^1,2^, many responses are being pursued in parallel, including infection prevention and control, disease surveillance, antibiotic stewardship, and the development of new therapeutics, including so-called “non-traditional” therapeutics.

Although there is no universal definition of “non-traditional,” Tse et al.^3^ define traditional products to include “small-molecule agents that directly target bacterial components to exert a bacteriostatic [i.e., growth prevention] or bactericidal [i.e., killing] effect,” whereas non-traditional agents include “antimicrobial therapeutics that work through other means (i.e., not a small molecule and/or utilizes a non-traditional target).” Non-traditional agents are diverse, including agents that modify the microbiome, chelators that inactivate the metals needed for bacterial enzymatic activity, nucleic acids that interfere with bacterial DNA, and antibodies or viruses (bacteriophages) that target microorganisms or their toxins with high specificity (Table 1). Their diversity reflects radically different mechanisms and approaches that may improve treatment options and, in theory, delay the development of resistance.

**Table 1:** The wide range of non-traditional antimicrobial agents

Category	Essential mechanism (breadth of effect)^a^	Examples of approved^b^ products
Antibiotic-sequestering products or antibiotic-degrading enzymes	Physical binding or destruction of antibiotic molecules that reach the large bowel, thereby limiting damage to the microbiome and reducing risk of *Clostridium difficile*-related diarrhea (Broad) and the spread of antibiotic residue in the environment	No approved examples
Antibodies	Inactivation of a pathogen, a virulence factor, or a toxin (Narrow)	Antisera to the toxins that produce the clinical syndromes of anthrax, diphtheria, botulism, and tetanus.
Bacteriophage (both wild-type and engineered)	Direct lysis of target bacteria (Narrow)	No approved examples
Host immune response modifiers (stimulating and immunosuppressive)	Augmenting or suppressing host immune response to modify course of infection (Broad)	Interferon-gamma, G-CSF
Lysins	Direct lysis of target bacteria (Narrow)	No approved examples
Metal chelation	Inactivation of key bacterial enzymes by chelation of zinc, manganese, or iron from the bacterial enzyme (Broad)	No approved examples
Microbiome and probiotics	Modification of microbiome to eliminate or prevent carriage of resistant or pathogenic bacteria (Narrow to broad)	No approved examples
Nucleic acids, antibacterial (CRISPR and related)	Anti-sense or target destruction used to interfere with bacterial DNA (Narrow)	No approved examples
Nucleic acids, anti-resistance	Direct killing of bacteria by nucleic acids (Narrow)	No approved examples
Peptides, antibiofilm	Peptides based on innate defense peptides (defensins) or other sources may exhibit direct antibacterial effects (Broad)	No approved examples
Peptides, antimicrobial		
Peptides, innate host defense		
Toxin sequestration or removal	Removal of bacterial toxins may modify the course of infection (might be Broad or Narrow)	No approved examples
Vaccines	Prevention of infection by induction of an antibody response that interferes with bacterial pathogenesis (Narrow)	Many examples (e.g., vaccines for *S. pneumoniae*)

^a^Breadth of effect: Narrow = activity is usually limited to a single species of bacteria and hence likely to require targeting via a diagnostic; Broad = breath of activity might be sufficiently broad to permit empiric use against the typical range of bacteria causing a given syndrome. ^b^Approved in the US or EU. (Categories adapted from refs. 5 and 3)

Non-traditional therapeutics have been the subject of recent reviews^3–6^, symposia (22 April 2018, European Congress of Clinical Microbiology & Infectious Diseases, http://amr.solutions/blog/eccmid-symposium-expediting-antibacterial-development-core-lessons-and-key-tools-for-a-rocky-road), and workshops (14 June 2018, Duke-Margolis Center for Health Policy, https://healthpolicy.duke.edu/events/understanding-development-challenges-associated-emerging-non-traditional-antibiotics; US Food and Drug Administration, 21–22 Aug 2018, https://www.fda.gov/drugs/newsevents/ucm606052.htm). A common theme has been the question of how non-traditional products can best be developed. In these discussions, questions have been raised about whether existing approval pathways are adequate, or whether there is a need for new regulatory approaches to better facilitate the development of novel drugs that for reasons discussed below would find it difficult to generate data adequate to support approval.

In previous decades, such questions would have been raised by large global pharmaceutical companies using their extensive resources to advocate for efficient development and approval standards. But today, as recognized by numerous authors^7–10^, most larger companies have discontinued their antibiotic development programs, including recent exits by Novartis, AstraZeneca, Sanofi, Allergan, and The Medicines Company^11,12^.

As a result, most companies currently pursuing preclinical antibiotic programs are small, entrepreneurial endeavors^13^. Many are in the CARB-X portfolio (https://carb-x.org/), a government- and charitable foundation-funded non-profit project launched in 2016 and tasked with investing up to $500 million in very early-stage antibiotic projects around the world. Since smaller companies generally lack the resources to effectively advocate for their collective needs, two authors of this paper who have been involved with creating and running CARB-X (J.H.R. and K.O.) felt it was important to address pressing development issues to ensure that the regulatory infrastructure is prepared to efficiently evaluate these new technologies and advance them through development and approval.

In this Perspective, we conclude that (a) the idea of a non-traditional product has only limited relevance to regulatory decision-making; (b) most products classified as non-traditional can and should be developed within the existing regulatory framework using assessment measures based on how the therapeutic agent affects the way the treated individual feels, functions, or survives; and (c) there are innovative product types with primarily population-level and future benefits that may warrant further discussion regarding how these benefits can best be incorporated into the drug evaluation paradigm.

## The idea of “non-traditional” has limited utility

The definition by Tse et al.^3^ includes as non-traditional both products with non-traditional properties (e.g., lacking intrinsic antibacterial activity on their own and hence neither bactericidal nor bacteriostatic) as well as antibacterial products that are not small molecules. This definition can be improved by disaggregating it into its two components: non-traditional *molecular structures* and non-traditional *product goals* (Table 2).

**Table 2:** Two categories of traditional (T) vs. non-traditional (NT)

	Traditional (T)	Non-traditional (NT)
Structure	Typical small molecule	Bacteriophage, lysins, (monoclonal) antibodies, charcoal, and oligonucleotides
Development goal	Treatment or prevention of a standard infection	Other goals, such as prevention of development/acquisition of resistance, improving/restoring microbiome status, and slowing the spread and resistance in the population at large

Distinguishing traditional and non-traditional *molecular structures* separates small-molecule antibiotics from larger molecules or radically different products. Typical small-molecule drugs are completely characterized chemically, and usually have a molecular weight less than ~1000 Da. Non-traditional products such as bacteriophages or genetically engineered antibodies are usually much larger and sometimes not readily visualized without the aid of computer modeling. In some cases, the product is a mixture of substances that cannot easily be described except by reference to functional characteristics (e.g., polyvalent antisera characterized by ability to neutralize a target). There are also non-traditional products that are radically different, such as charcoal-based absorbents that selectively sequester antibiotics in the lower gastro-intestinal tract to reduce disruption of healthy intestinal flora^14^, or lipid-based products that absorb bacterial toxins without directly affecting bacterial growth^15^.

The idea of traditional vs. non-traditional *product goals* offers a different comparison. Traditional goals encompass prevention (vaccines) and direct effects on bacterial growth, including killing of bacteria (therapeutics). By contrast, non-traditional goals, include reducing the impact of bacterial toxins; hindering the formation of biofilm, a dense, extracellular matrix produced by some microbes that can be difficult for antibiotics or the immune system to penetrate; preventing colonization with organisms that could propagate antibiotic resistance genes; and improving/restoring microbiome diversity. As with traditional antibiotics, which both treat the individual and simultaneously reduce risk of transmission to others, new antibiotics aimed at non-traditional goals may produce both individual benefit (e.g., if an individual does not become colonized with *Salmonella*, then infection due to *Salmonella* would only occur if initial exposure led immediately to infection), and also benefit to others (e.g., if an individual is not colonized with *Salmonella*, then transmission to others via environmental shedding is not possible)^16^. But unlike traditional products intended for and developed based on measures of individual benefit, some products are being developed primarily for their population-level or future benefits.

Whereas non-traditional structures and some non-traditional goals are adequately served by current regulatory paradigms, progression of products with non-traditional goals focused on population-level benefits may require extension of current paradigms, as discussed below.

### Four instructive development categories (STAR)

**Fig. 1:**
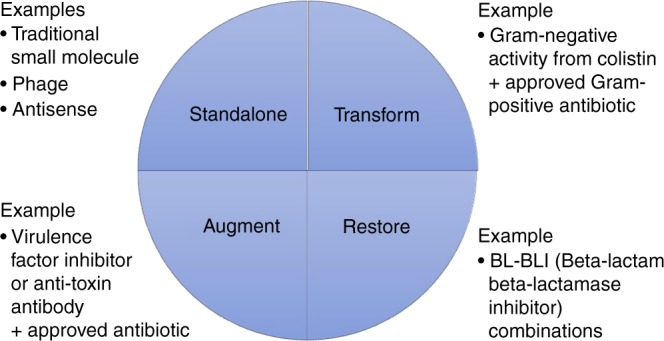
STAR: The four novel product categories. Both traditional and non-traditional antibiotic products can be placed into one four fundamental categories: Standalone (effective as monotherapy), Transform (extends the range of activity of an existing product), Augment (enhances the effect of an otherwise effective product), and Restore (rejuvenates the activity of an antibiotic that otherwise lost utility)

The value of a new antibiotic product is determined by its ability to achieve a particular therapeutic outcome or to achieve public health goals; it is not dependent on its structural classification as traditional or non-traditional. To see this, categorizing both traditional and non-traditional antibiotic products into four development archetype categories of Standalone, Transform, Augment, and Restore (STAR, Fig. 1) helps identify relevant regulatory and ethical considerations:

- *Standalone:* Products in this category are effective as monotherapy. Essentially all traditional small molecules fit here, but direct-acting bactericidal products with non-traditional structures (e.g., bacteriophage) could also be standalone. Vaccines will generally fit here.
- *Transform:* The product transforms (or extends) the range of an existing product to enable action against microorganisms not previously susceptible to the existing product. For example, efforts are underway to use polymyxin B analogues to allow drugs currently effective only against Gram-positive bacteria to enter and act on Gram-negative bacteria as well^17^. To date, there are no approved products in this category.
- *Augment:* The product augments or improves on the activity of an otherwise active and effective antibiotic. Products seeking to reduce bacterial virulence or neutralize a bacterial toxin are an example of this category and have to date been polyclonal or monoclonal antibodies^18,19^. Approved examples of this category are limited to a small number of products that neutralize toxins in diseases where the toxin (rather than the bacterial burden) is the primary cause of the clinically apparent disease. Examples include a monoclonal antibody (raxibacumab) to a toxin of *Bacillus anthracis*^20^, a polyclonal equine antiserum for toxins of *Corynebacterium diphtheriae*^21^, antisera for toxins produced by *Clostridium botulinum* (polyclonal equine and polyclonal human antisera)^22^, and a polyclonal human antiserum for toxins produced by *Clostridium tetani*^23^. With these products, a standard antibacterial is often used to eliminate carriage of the toxin-producing organism and thereby both stop production of toxin and reduce transmission to others of the organism, but the anti-toxin antibodies have a strong activity independent of clearance of the bacteria. In a related but not identical fashion, a monoclonal antibody to a toxin of *C. difficile* (bezlotoxumab) also exists but its primary effect is to reduce recurrence following control of active disease by a standard antibiotic therapy^24^.
- *Restore:* The product restores the activity of an existing product that has lost utility due to development of resistance (e.g., evolution allowing the bacteria to produce beta-lactamase, an enzyme that breaks down beta-lactam antibiotics to render them ineffective). Several products in this category have been approved (e.g., amoxicillin combined with the beta-lactamase inhibitor clavulanic acid).

Note that three of these categories (*Transform*, *Augment*, and *Restore*) are intrinsically combinations in which the novel agent also could be described as a potentiator, enhancer, or adjuvant of the product with which it is paired. These terms are non-specific, however, and have been applied interchangeably^25^. The terms *Transform*, *Restore*, and *Augment* better distinguish the function of the various types of products currently being developed, and are therefore preferable.

### Four development categories: implications

Products in all four of the STAR categories can be developed using current regulatory approaches for establishing non-inferiority or superiority (see Box 1), although problems of dose selection (Box 2) and patient enrollment may still occur.

While superiority trials provide stronger evidence of efficacy, in many infection settings the only plausible trial design is non-inferiority (Box 1). In particular, non-inferiority trials are likely appropriate for the *Standalone, Transform, and Restore* categories (Table 3). In trials of these types of agents, the new agent will be compared to an existing control agent in the so-called usual drug resistance (UDR) setting^26^ in which resistance is not extreme and one or more reliably active control agents can be used as standard-of-care comparators. The UDR setting is used because although preclinical evidence may suggest that the novel therapy would offer incremental therapeutic benefit specifically in patients harboring resistant bacteria, it would be unethical to randomize such patients to a control agent known or predicted to be ineffective due to resistance, particularly where there is a substantial risk of morbidity or mortality^27,28^. Yet in the UDR setting, the fact that the control is a therapy that is safe and highly effective in curing most patients in the population makes it unlikely that a new product could demonstrate superiority—what is superior to achieving patient cure?

**Table 3:** Development options for the four categories

Design options	Would data from a non-inferiority study be adequate for approval?^a^	To what extent is a demonstration of superiority required for approval?^b^
Standalone	Yes	Optional
Transform	Yes	Optional
Augment	No	Required
Restore	Yes	Usually optional

^a^Is it possible to achieve initial approval by studying the product in a head-to-head non-inferiority study in which the novel product is compared with an existing agent in a usual drug resistance (UDR) setting where the comparator agent has retained activity?

^b^Recognizing the conflicting tension around use of superiority studies for approving new agents (see text), does demonstration of the value of the novel agent effectively require a superiority study?

Developers of new drugs for resistant pathogens thus face a conundrum. Ideally, they would develop data on the new product in the face of resistant pathogens before the onset of a widespread public health concern. But until such an outbreak, there will be at least one existing reliable agent for the UDR bacteria currently extant. That such reliable agents exist is desirable for society, of course, but what it demands is that infected patients be treated either with a reliable agent or something in equipoise with it. Even in those worrisome scenarios in which there is no currently reliable agent for UDR, the development of the first new product that addresses resistance resets the standard of care, as the new agent becomes the new reliable intervention.

The result is that in the (desirable) presence of a reliable agent for current UDR bacteria, development of new antibiotics must rely on non-inferiority design in the UDR setting. Although the pivotal UDR trial does not directly establish evidence of effectiveness in populations for which existing antibiotics are less effective or ineffective, two lines of logic suggest the new agent is likely to be effective in a future setting when currently reliable agents fail. First, and generally limited to agents acting by a mechanism different from that of existing agents, emergence of resistance to the currently reliable comparator will usually have no impact on the microbiologic activity of the new drug: the new drug is expected to retain the activity demonstrated in the UDR study whether or not the older drug retains its activity (i.e., cross-resistance is unlikely). Second, data in second-line non-UDR settings in which older agents have failed can often provide at least a small-scale demonstration of the potential of the new compound.

While, for the reasons discussed, non-inferiority designs are often necessary for products in the *Standalone, Transform*, and *Restore* categories, a demonstration of superiority becomes preferable and perhaps required for *Augment* and sometimes for *Restore* (as discussed below). To understand this, consider a hypothetical non-traditional product “AUGMENT” designed to enhance immune control of an infection when given with “EXISTING,” a currently approved antibiotic. Regulations generally require that each component of a fixed-combination product contribute to its claimed effects, such as enhancements to the safety or effectiveness of the principal active component^29^. A demonstration of similar efficacy (non-inferiority) between two trial arms would fail to meet this requirement: only by showing that AUGMENT + EXISTING *improves* on EXISTING is it demonstrated that the new component of AUGMENT contributes to the product’s claimed effects.

Given the high (usually ≥ 80–90%) success rates with modern antimicrobials, the requirement to demonstrate further increased efficacy is a very challenging hurdle, although a new drug could correct some defect in an older one. For example, AUGMENT might offer incremental value by simplifying dosing or reducing the side-effects of EXISTING as some formulation improvements of traditional antibiotics have done. But absent a plausible additional clinical benefit, *Augment* developers will struggle to justify reasons to undertake clinical trials.

A hurdle similar to that for *Augment* may also occasionally arise for *Restore*. Consider a novel beta-lactamase inhibitor (BLI) that protects a beta-lactam (BL) antibiotic from destruction by beta-lactamase enzymes. Similar to the problem discussed for AUGMENT + EXISTING, the value of BLI + BL combination is only seen when the infecting organism is resistant to the BL but susceptible to the BLI + BL. If it is easy to find such organisms, then a study of BLI + BL vs. an existing product of a different class that has retained activity (usually a carbapenem, the typical reliable drug of current development programs for UDR Gram-negative bacteria) will show the BLI + BL to work when the BL alone would have lacked activity. If, however, the relevant bacteria are very rare, it may be almost impossible to show the advantage of the combination.

Non-inferiority designs can therefore be used to evaluate products in the *Standalone*, *Transform*, and *Restore* categories without waiting for resistance to develop such that effective therapy is unavailable (Table 4). Although the primary benefit of such products derives from their theorized abilities to address future resistance, perhaps as demonstrated by laboratory or animal data, the non-inferiority clinical design means that robust evidence of effectiveness in such future human populations is not available at the time of approval.

**Table 4:** Why superiority is pursued in some areas but not routinely available for anti-infectives

Why not superiority?	Migraine	Cancer	Infection
1. Durable cure is routine	No	No	Yes
2. Placebo is routinely acceptable	Yes	No	No
3. Transmissible resistance arises, thus new agents always needed	No	No	Yes
4. New agents are really for use…	Today	Today	Tomorrow

In the table, the constraints for developing new agents for migraine, cancer, and infection are considered. For migraine, delayed treatment is painful but has no long-term consequences, and thus it would be ethical to ask a study participant to enroll in a trial where one arm was only placebo (or delayed therapy). In the case of cancer, placebo is not acceptable but durable cure is not routine and there remains substantial opportunity for improvement on current therapies. For anti-infectives, however, durable cure is expected as routine and placebo (or deliberate use of ineffective therapy) is not ethical if there is any alternative whatsoever. The use of non-inferiority designs in infection offers a way to proactively address the need to produce new agents to address emerging resistance and to complete such development programs *before* resistance is sufficiently widespread that the current agents are frequently ineffective

### Normal regulatory requirements apply to non-traditional products

Given their intriguing potential properties (Table 1), it is sometimes suggested that non-traditional products could perhaps be exempt from the normal regulatory approval rules. However, there is typically no reason for such products to be exempt from the usual requirements to demonstrate safety and efficacy. For example, if an effect on a clinical outcome cannot be used, efficacy could perhaps be established by showing an impact on a surrogate outcome measure (such as microbiome change). But, such an outcome measure would be held to the standard of “reasonably likely to predict clinical benefit” under the FDA’s accelerated approval pathway^30–32^. Unless such standards for surrogate markers of efficacy are met, evidence of impact on a novel endpoint might be hypothesis-generating but could not be used to support approval.

Box 1 Non-inferiority vs. superiority trial designs All pharmaceutical products must (a) show which individuals can benefit from the product, (b) demonstrate a way to identify those individuals, and (c) document the benefit received from the product. Clear answers to these questions are required for product approval and acceptance. To achieve this, one of two types of trial designs can be used^55–59^. In a superiority trial, the goal is to show that a product, “NEW,” is measurably superior to “EXISTING” or to a placebo. In a non-inferiority trial (sometimes formerly referred to as an “equivalence trial”), the goal is to show that NEW has efficacy similar (within the bounds of a pre-specified non-inferiority margin) to that of EXISTING. Each design has strengths and weaknesses.

Superiority trials are more compelling and are preferred whenever possible. Because they demonstrate that NEW improves on EXISTING (or on placebo), a positive outcome shows simultaneously: (1) the advantage of NEW, and (2) that the group of individuals are likely to benefit from NEW can be identified.

In contrast, a non-inferiority demonstration of similar outcomes between NEW and EXISTING could mean either that (a) NEW and EXISTING both indeed had similar benefits subject to the non-inferiority margin and statistical limits of the study, or (b) NEW and EXISTING both did nothing and hence appear to have similar efficacy, which could occur, for example, if inadequate screening led to the enrollment of subjects with viral pneumonia in a trial intended to test two treatments for bacterial pneumonia. In practice, there are many ways that non-inferiority trials can produce flawed results^28,59^.

Although superiority trials would thus seem the natural choice, the need to show superiority becomes a weakness in the setting of bacterial infections given their acute onset, high morbidity and mortality risk, lack of timely precision diagnosis, and the importance of immediately beginning a reliable therapy^27,28^. Except for trivial situations, acute infections are associated with striking risks when not treated promptly and properly. It is thus imperative to offer effective therapy if at all possible. However, when a safe and highly effective therapy serves as the comparator in a trial, it is unlikely that a new product would more effectively clear an infection than the comparator. Furthermore, efforts to control the spread of drug-resistant infections make it more difficult to enroll the patients needed for a superiority design trial. While it might be hoped that modern molecular diagnostics could solve this problem, practical considerations reduce their impact. Consider, for example, an uncommon bacterium of interest (perhaps a specific species or perhaps a form of resistance) that is seen in only 1% of cases of a given syndrome. Even if diagnostics could rapidly identify cases of interest, 100 cases must be screened to find a single case of interest. As diagnostic tests never have perfect sensitivity or specificity, the required number to screen would be even higher.

The speed with which most infections progress also works against case finding. Unlike rare genetic disease or tumors where there is time to refer to a specialty center, acute infections progress to produce substantial morbidity and mortality over hours to a few days. It is standard practice to initiate “empirical therapy” within a few hours of diagnosis, that is, treatment with an antibiotic expected to be effective based on a clinician’s educated guess as to the identity of infectious agent, before the particular pathogen has been more robustly identified with a diagnostic test. With the exception of a small number of chronic bacterial infections, this means that the patient with the rare bacterium must present at a facility already running the relevant trial. If empirical therapy proves adequate to eradicate the organism, the ability to demonstrate the activity of the test product is further diminished.

Thus, superiority designs work ethically and methodologically only where there is truly no proven alternative. This may be the case in truly grave public health circumstances involving resistant pathogens, but once a novel effective therapy is created, superiority designs again become difficult.

In contrast, non-inferiority designs allow trials to proceed without requiring resistance to develop to the point that effective therapy is widely unavailable. The real value of the new agent resides in its theorized but not yet fully proven utility to future patients (Table 4 and ref. ^59^).

Box 2 Dose selection for products lacking direct antimicrobial activity An important challenge could arise for any of the four categories depending on the product. Antibiotic development relies heavily on dose selection based on prediction from animal models of the antibiotic exposures (pharmacokinetics (PK) and pharmacodynamics (PD)) likely to be efficacious^60,61^.

Traditionally, the concept of minimum inhibitory concentration (MIC) has been central to the mathematical underpinnings of PK-PD. The MIC is the minimum concentration of a drug that prevents visible growth in a laboratory test tube or Petri dish. It is also used as a key element when determining the drug concentration levels required to obtain an effective response^62^. Given the extensive experience with MIC-based PK–PD to predict efficacy^63,64^, a demonstration that efficacy should follow from a given drug concentration^65^ can support approval of an antibacterial agent based on a single-clinical trial^28^.

Some non-traditional products in development lack an MIC, such as agents designed to absorb bacteria-produced toxins while leaving the bacteria themselves unaffected. A compelling demonstration of clinical activity for such products may require more clinical data than for products with supportive MIC-based PK–PD evidence. This challenge might be less for products designed to lower the MIC of an existing drug when part of a combination^25,66^.

## Non-traditional goals: the issue of population-level benefits

Traditionally, antimicrobial products have been developed to provide direct benefits to the individuals receiving treatment. Although these products have always produced positive externalities in the form of reduced opportunities for transmission to third parties^33^, companies have recently begun to develop products designed to benefit third parties while offering only occasional benefits to the treated individual.

For example, a product may focus on preventing colonization (and hence seeking to reduce the risk of infection) rather than treating clinical symptoms after invasive infection develops. At a recent workshop on non-traditional products (https://www.fda.gov/drugs/newsevents/ucm606052.htm), participants considered such a hypothetical product: “Z-3,” postulated to prevent acquisition of Metallo-Beta-Lactamase-producing (MBL-producing) Gram-negative bacteria in gut flora. MBL is an enzyme that can degrade certain antibiotics, including carbapenems, and bacteria producing it are considered highly antibiotic-resistant. By preventing acquisition of MBL-producing bacteria, Z-3 could both reduce the risk of an infection due to such an organism as well as minimize opportunities for the MBL gene to spread.

As there are relatively few reliable agents for treating infections due to MBL-producing bacteria, the second benefit (reduced transmission) has public health implications. Although Z-3 would not prevent infections associated with gut flora such as UTI and appendicitis, such infections should not be due to MBL-producing bacteria but rather to less difficult-to-treat strains. Thus, Z-3 may offer some benefit to the treated person, but its potentially greater value comes from the public health benefit to others through reduced transmission of the difficult-to-treat bacteria.

Measuring those public health benefits is challenging. In traditional vaccine trials, an agent’s value is measured by reduction in observable clinical infections directly in the individuals receiving the vaccine. This effect is sometimes but not always mediated in part by preventing colonization^34,35^. In the case of Z-3, the major emphasis would be on preventing colonization with MBL-producing bacteria and extrapolating from lowered colonization rates to predict both individual and population-level clinical benefit, as clinical infections caused by non-MBL-producing bacteria in individual patients may not be reduced. Preventing colonization with MBL-producing bacteria by Z-3 could offer some benefit to treated individuals by reducing the risk of developing invasive MBL-producing bacterial disease, but the individual benefit might be quite small due to the infrequency of such colonization and the fact that, even if colonized, the individual might not develop invasive disease.

It might be possible to resolve these difficulties by developing and validating a surrogate marker, but that is not an easy task and the most obvious surrogate (lack of detectable colonization with resistant strains) is not acceptable due to the inability to demonstrate that it is reasonably likely to predict clinical benefit. In studies to date, the linkage between elimination of carriage and subsequent disease has been inconsistent^36,37^, presumably both because it is technically difficult to prove absence of carriage in every possible nook and cranny of the human body, and because pathogens can be acquired at any time from new environmental exposure. Thus, a 2013 guidance from EMA^38^ states that “Indications that relate to the reduction or eradication of a pathogen from a specified body site are not acceptable unless the microbiological effect of active treatment has been shown to result in a measurable clinical benefit.” To our knowledge there is no exact parallel statement in FDA guidance, but the need for a surrogate marker to convincingly predict a clinical outcome is apparent in FDA-supported documents that discuss degrees of validation of endpoints^31,32^ as well simply having strong face validity.

This leads to a significant challenge when the primary benefit of a proposed product accrues only in a much larger population than those directly treated. This raises important questions about how pivotal trials for a product like Z-3 should be designed, and to what extent regulators should take into account positive externalities that benefit both current and future populations not receiving direct interventions in the clinical study^33^. These products also pose challenges related to market demand that may prove to be more difficult to overcome than the regulatory considerations.

## Research ethics

In parallel to the scientific and regulatory issues that have been discussed so far, there are also ethical considerations that must be addressed.

First, and most fundamental, is the question of whether it is ethically acceptable to carry out non-inferiority trials when developing new antimicrobial agents. Outside a trial, those who might be asked to enroll would be treated with a reliable drug, i.e., an existing effective agent (assuming their infection is not resistant to all available therapies). Within the trial, they would be randomized either to the novel intervention or the reliable standard-of-care drug. However, in a non-inferiority trial, that novel intervention is not hypothesized to leave the participant better off than he or she would have been outside the trial, given that superiority is not expected. Instead, the only hypotheses are that participants randomized to the intervention arm of the trial could do as well as they would have done outside the study (best case) or potentially worse. Equipoise may still be satisfied in this scenario, as the expectation is that the novel intervention will be an acceptable clinical substitute for the established older drug such that no participant is made knowingly worse off as a result of randomization^39^. Nonetheless, except for those individual participants who turn out to be infected with a resistant pathogen, enrollment entails an entirely risk-based proposition compared to accepting standard care outside the study.

Non-inferiority trials have been criticized on these grounds, particularly on the basis that it is inappropriate to ask participants to accept such risks for the moderate (or even low) social value traditionally associated with non-inferiority design^40,41^. These criticisms are inapposite here, however. Unlike non-infectious disease products approved based on non-inferiority trials, new antibiotics approved based on such trials offer the promise of substantial social value if they are believed, based on non-clinical evidence, to be able to address future resistant infections (potentially, including those occurring in current study participants). Moreover, even if the new intervention is somewhat inferior to the proven effective current product, it could still be a useful treatment option in the face of future resistance when the current product is no longer effective.

Thus, despite their non-inferiority designs, these trials are not focused on *current non-inferiority*, but rather *future superiority* (Table 4). Although it may be important to take steps to reserve new interventions for use only in the future as a matter of stewardship, that is for regulators, prescribers, and payers to consider^42,43^; it does not dictate whether the study itself evaluates a worthwhile clinical question.

Hypothetically, it would be ideal to instead conduct superiority trials exclusively in patients with infections resistant to available therapies, since they would potentially be able to derive direct clinical benefit from study participation and the resulting data would be stronger. However, as noted above, the logistics of such a design may be impractical until such point as the fully drug-resistant bacteria are endemic (see Box 1), which would represent a global public health calamity. In other words, it would be unethical to wait to develop novel antibiotics until resistant diseases become more widespread—and it is only at that point that superiority trials might be feasible.

For these reasons, if we conclude that these are new products worth having because they have sufficient social value^44^, the impracticability of superiority designs and the fact that much of what is worrisome about non-inferiority designs is absent in this context means that non-inferiority designs may be acceptable.

But the question remains: is it justified to ask patients facing serious infection to risk treatment with an investigational product in a non-inferiority trial when they likely would be adequately treated by a reliable drug in the UDR setting and are therefore not likely to be made *better* off by participation? In other words, is equipoise—which focuses on the avoidance of *inferior* treatment—sufficient? The short answer is yes. This is because there is no ethical requirement that research have the potential to make participants better off compared to how they might fare outside a study, or even to offer them any possibility of direct benefit at all. If trials posing risks in the absence of the potential for superior or direct benefit were necessarily unethical, it would be impossible to conduct Phase I trials in healthy participants—or any non-therapeutic research. Instead, ethical obligations to study participants demand minimizing the risks to which they may be exposed, ensuring that remaining risks are justified by potential benefits, and protecting participant autonomy by securing adequate informed consent^45,46^. Each responsibility could be satisfied in this context.

First, if a non-inferiority study was designed so that (a) the dose of the novel agent is chosen to maximize its likely efficacy based on preclinical data and (b) individuals failing either study arm are promptly moved to a salvage therapy, the risk of inadequately treated infection or rapid progression would be addressed and the risk of serious harm to study subjects minimized. Thus, it would be possible to comply with the ethical standard espoused in the Good Clinical Practice guidelines (Point 2.3 of ICH E6 (R1)) that the “rights, safety, and well-being of the trial subjects are the most important considerations and should prevail over interests of science and society.”

Second, risks to individual trial participants are often justified by the prospect of benefit that would accrue only or primarily to future patients^44,47^. Indeed, Miller and Joffe^39^ argue that “risk–benefit assessment, geared to the purpose of clinical trials in developing knowledge to inform health-policy decisions for populations of patients,” is more important to assessing a trial’s ethical permissibility than the presence of equipoise as traditionally understood. Moreover, U.S. regulations governing research with human subjects explicitly acknowledge that study risks and benefits may not necessarily accrue to the same individuals, permitting Institutional Review Boards to approve proposed research when “[r]isks to subjects are reasonable in relation to anticipated benefits, *if any*, to subjects, and the importance of the knowledge that may reasonably be expected to result” (emphasis added)^45,46^.

Of course, this can be a challenging calculation for research ethics review committees, especially considering that there is no well-accepted upper threshold of permissible research risk^48^ and reasonableness standards are notoriously fuzzy. It can be facilitated, however, by using “component analysis,” which distinguishes between research procedures that offer the prospect of direct clinical benefit to participants and those that are non-therapeutic^49^. In the context of a non-inferiority trial of a novel antibiotic, infected participants are offered the prospect of direct benefit and expected to fare as well as they would in standard care, while the risk that they will not—if minimized as described above—is likely justified by potential public benefits of having a new effective therapy available in the armamentarium against resistant bacteria. Thus, we anticipate that this type of research can easily satisfy required risk–benefit analysis.

Finally, it is important to explain to potential participants that they could be treated outside the trial with an agent predicted to be safe and effective, absent resistance, and that by enrolling they would face risk predominantly for the benefit of others^41,50–52^. Some may be willing to do this altruistically and some may need financial incentives, but both can be acceptable motivations^53,54^. Assuming there is reason to believe there would be enough willing participants to adequately enroll a trial—an essential ethical assumption, as otherwise the risks to participants could not be outweighed by potential social benefits because it would not be possible to answer the study questions—non-inferiority designs to develop new interventions to gird the future stock of available therapeutics can satisfy essential ethical criteria.

It is essential to note, however, that the social benefit that is ethically required of research is not guaranteed simply by developing a theoretically useful novel antibiotic. If patients would not have adequate reason to take that product once approved, the potential for social benefit will be dramatically limited, raising a range of practical and ethical concerns preliminary to any questions regarding appropriate regulatory pathways for approval.

Consider again the hypothetical product Z-3, theorized to prevent human acquisition of non-pathogenic MBL-producing bacteria. Prevention typically depends on patients reasonably anticipating the implications *for them* of clinical infection by a pathogen. For example, unless they are required to be vaccinated by their employer, people are likely to receive the influenza vaccine because they anticipate they are likely to be exposed to the virus and do not wish to suffer from infection. From the individual perspective, it is often only an incidental benefit that their contacts may also be protected by their vaccination.

For a product like Z-3 to be taken up in practice, individuals would have to anticipate a sufficient individual benefit stemming from a reasonable likelihood of clinical infection due to MBL-producing bacteria. Otherwise, they are not likely to use a product to prevent such infection simply for the public health benefit to others. Although state governments have the authority to impose public health measures (e.g., preventative interventions such as vaccination), such authority is permissible only for compelling and direct public health benefits of a sort not likely for a product like Z-3. Therefore, use of the product will have to be motivated by patients themselves rather than some external mandate. Perhaps individuals living in or visiting certain countries in which bacteria susceptible to Z-3 are more prevalent would have adequate concerns to motivate its use for their own benefit, but they would have to be convinced that the risks are sufficiently great that they will become infected and suffer clinical sequelae from such bacteria, which is not something that can be taken for granted.

Clinical trials to evaluate a product like Z-3 would be ethically acceptable only if there is an adequate population likely to utilize the product once approved and marketed. As described above, such trials would otherwise subject participants to risks and burdens that could not be justified by their ostensible social value. Note that this stands in contrast to novel antibiotics intended to treat individual patients when they lack other options for quelling a current infection with resistant bacteria; these patients would clearly have reason to take such a drug for their own benefit, if approved.

Assuming there is a plausible patient population and path to approval for Z-3, along with sufficient numbers of willing participants for adequate clinical trials, the primary ethical considerations arising for trial participants would be risk minimization, adequate balance of risks and potential benefits to subjects and society, and informed consent, as discussed above with regard to non-inferiority design. However, the questionable clinical utility and acceptability of a product like Z-3 is likely to be the most substantial hurdle to its development, due to the modest direct benefits to the patient and difficulties creating a market for more diffuse benefits to public health. Designing a trial that can capture both the direct and population-level benefits of a product like Z-3 will not be simple and may require techniques such as cluster randomization to allow measurement of the population benefits of lower resistance rates in, for example, hospitals using vs. not using the product. Approval pathways could also be challenging given measurable benefits primarily at the population rather than individual level, but this is a secondary concern behind the development issues related to an uncertain market for these products and associated ethical concerns for justifying clinical trials.

## Summary

The idea of non-traditional antibiotic products has generated substantial interest because of the potential for radically different mechanisms of action that could address unmet needs. These products have potential value for society, but investment in these products may be suboptimal if the regulatory requirements for demonstrating safety and efficacy are not clear.

Developers of non-traditional antibacterial agents should not expect to bypass established regulatory and ethical standards. Rather, most non-traditional products fall readily into one of the four arms of the STAR paradigm, and can be tested using existing non-inferiority- or superiority-based trial designs under existing regulations applicable to traditional small-molecule products.

While most antibiotics are currently approved based on non-inferiority designs^27,31^, some non-traditional products can best (or must) show their value via a demonstration of superiority over existing products. This challenge is most evident in the case of products in the *Augment* category where a partner therapy (or the antibacterial standard of care) has retained its activity, but where it is hoped that efficacy can be improved. Here, the core test is of the combination vs. the existing product alone, and the combination will be required to be superior. The existing product must be fully dosed and expected to yield its baseline level of efficacy. Given the good efficacy of current tools, superiority is predicted to be difficult to demonstrate. It thus seems unlikely that this pathway will be routinely feasible and there is no obvious solution for this problem — if the partner compound is highly efficacious, then simply showing similar efficacy in combination with an *Augment* agent does not provide a reason to use the new product in the absence of other information.

Turning to the question of non-traditional goals, the primary benefit in all the trials just discussed will be observable at the level of the individual treated with the new agent, in some cases based on newly validated surrogate endpoints. A challenge remains, however, for products with the primary potential for population-based benefits, including benefits to future generations. Although these may be important products from a public health perspective, if they are insufficiently attractive to the patients who would need to take them because they are insufficiently relevant to their individual health, development is likely to be stymied by practical and ethical issues even before the challenges of regulatory approval arise. For these products, it will be important to identify an appropriate clinical market in which individual benefit is sufficiently large to motivate use.

In summary, the idea of a non-traditional product has only limited relevance to regulatory decision-making as most products classified as non-traditional can and should be developed within the existing regulatory framework. That said, innovative product types with primarily population-level and future benefits can be envisioned and these would seem to warrant further discussion regarding how such benefits can best be incorporated into the drug evaluation paradigm.

## References

[1] Review on Antimicrobial Resistance. *Tackling Drug-Resistant Infections Globally: Final Report and Recommendations*. Available online at http://amr-review.org/ (2016 May). This final paper from a multi-year effort to review the economic challenges surrounding antimicrobial resistance provides a thorough review of the entire area.

[2] World Bank Group. *Drug-Resistant Infections: A Threat to Our Economic Future*. http://documents.worldbank.org/curated/en/323311493396993758/pdf/final-report.pdf (2017). Accessed 26 June 2019.

[3] Tse BN (2017). Challenges and opportunities of nontraditional approaches to treating bacterial infections. Clin. Infect. Dis..

[4] Opal SM (2016). Non-antibiotic treatments for bacterial diseases in an era of progressive antibiotic resistance. Crit. Care.

[5] Czaplewski L (2016). Alternatives to antibiotics-a pipeline portfolio review. Lancet Infect. Dis..

[6] Theuretzbacher, U. & Piddock, L. J. V. Non-traditional Antibacterial Therapeutic Options and Challenges. *Cell Host Microbe.* **26**, 61–72 (2019). 10.1016/j.chom.2019.06.004 31295426

[7] Outterson K. New business models for sustainable antibiotics. Chatham House. http://www.chathamhouse.org/publications/papers/view/197446 (2014). Downloaded 2 Mar 2014.

[8] Sertkaya, A. et al. Analytical framework for examining the value of antibacterial products. Report to US DHHS. United States Department of Health and Human Services. http://aspe.hhs.gov/sp/reports/2014/antibacterials/rpt_antibacterials.cfm (2014). Downloaded 17 June 2014.

[9] Ardal, C. et al. Developing new economic models to incentivise antibiotic discovery and development activities while safeguarding the efficacy of antibiotics by researching and advocating their appropriate use. http://drive-ab.eu/wp-content/uploads/2018/01/CHHJ5467-Drive-AB-Main-Report-180319-WEB.pdf (2018). Accessed 14 Oct 2018.

[10] Ardal C, Rottingen JA, Opalska A, Van Hengel AJ, Larsen J (2017). Pull incentives for antibacterial drug development: an analysis by the transatlantic task force on antimicrobial resistance. Clin. Infect. Dis..

[11] Kasumov A. Novartis Exits Antibiotics Research, Cuts 140 Jobs in Bay Area. Bloomberg News. https://www.bloomberg.com/news/articles/2018-07-11/novartis-exits-antibiotics-research-cuts-140-jobs-in-bay-area (2018). Downloaded 26 Nov 2018.

[12] Paton, J. & Kresge, N. Superbugs Win Another Round as Big Pharma Leaves Antibiotics. Bloomberg News. https://www.bloomberg.com/news/articles/2018-07-13/superbugs-win-another-round-as-big-pharma-leaves-antibiotics (2018). Downloaded 26 Nov 2018.

[13] Theuretzbacher U, Savic M, Ardal C, Outterson K (2017). Market watch: innovation in the preclinical antibiotic pipeline. Nat. Rev. Drug. Discov..

[14] de Gunzburg J (2018). Protection of the human gut microbiome from antibiotics. J. Infect. Dis..

[15] Laterre PF (2019). CAL02, a novel antitoxin liposomal agent, in severe pneumococcal pneumonia: a first-in-human, double-blind, placebo-controlled, randomised trial. Lancet Infect. Dis..

[16] Marineli F, Tsoucalas G, Karamanou M, Androutsos G (2013). Mary Mallon (1869-1938) and the history of typhoid fever. Ann. Gastroenterol..

[17] Corbett, D. et al. Potentiation of antibiotic activity by a novel cationic peptide: potency and spectrum of activity of SPR741. *Antimicrob. Agents Chemother*. **61**, e00200-e00217 (2017). 10.1128/AAC.00200-17 PMC5527571 28533232

[18] Sparrow E, Friede M, Sheikh M, Torvaldsen S (2017). Therapeutic antibodies for infectious diseases. Bull. World Health Organ..

[19] Wagner EK, Maynard JA (2018). Engineering therapeutic antibodies to combat infectious diseases. Curr. Opin. Chem. Eng..

[20] Head BM, Rubinstein E, Meyers AF (2016). Alternative pre-approved and novel therapies for the treatment of anthrax. BMC Infect. Dis..

[21] Huygen K (2016). Development of human monoclonal antibodies to diphtheria toxin: a solution for the increasing lack of equine DAT for therapeutic use?. Virulence.

[22] Anonymous. Investigational Heptavalent Botulinum Antitoxin (HBAT) to Replace Licensed Botulinum Antitoxin AB and Investigational Botulinum Antitoxin E. *MMWR Morb. Mortal. Wkly. Rep.* https://www.cdc.gov/mmwr/preview/mmwrhtml/mm5910a4.htm (2010). Accessed 26 June 2019. 20300057

[23] Blake PA, Feldman RA, Buchanan TM, Brooks GF, Bennett JV (1976). Serologic therapy of tetanus in the United States, 1965-1971. J. Am. Med. Assoc..

[24] Gerding DN (2018). Bezlotoxumab for prevention of recurrent clostridium difficile infection in patients at increased risk for recurrence. Clin. Infect. Dis..

[25] Wright GD (2016). Antibiotic adjuvants: rescuing antibiotics from resistance. Trends Microbiol..

[26] McDonnell AM (2016). Efficient delivery of investigational antibacterial agents via sustainable clinical trial networks. Clin. Infect. Dis..

[27] Rex John H, Talbot George H, Goldberger Mark J, Eisenstein Barry I, Echols Roger M, Tomayko John F, Dudley Michael N, Dane Aaron (2017). Progress in the Fight Against Multidrug-Resistant Bacteria 2005–2016: Modern Noninferiority Trial Designs Enable Antibiotic Development in Advance of Epidemic Bacterial Resistance. Clinical Infectious Diseases.

[28] Rex JH (2013). A comprehensive regulatory framework to address the unmet need for new antibacterial treatments. Lancet Infect. Dis..

[29] Code of Federal Regulations. 21 C.F.R. § 300.50 (“Fixed-combination prescription drugs for humans”), https://www.accessdata.fda.gov/scripts/cdrh/cfdocs/cfcfr/cfrsearch.cfm?fr=300.50 (2011).

[30] Food and Drug Administration, Center for Drug Evaluation. Labeling for Human Prescription Drug and Biological Products Approved Under the Accelerated Approval Regulatory Pathway—Guidance for Industry. *US Dept HHS, FDA, CDER, CBER.* http://www.fda.gov/Drugs/GuidanceComplianceRegulatoryInformation/Guidances/default.htm (2019). Accessed 10 Feb 2019.

[31] FDA-NIH Biomarker Working Group: BEST (Biomarkers E, and other Tools) Resource. Reasonably Likely Surrogate Endpoint. Food and Drug Administration (US) / Co-published by National Institutes of Health (US), Bethesda (MD). https://www.ncbi.nlm.nih.gov/books/NBK453485/ (2017). Accessed 6 June 2019.

[32] Biomarkers Definitions Working Group. (2001). Biomarkers and surrogate endpoints: preferred definitions and conceptual framework. Clin. Pharmacol. Ther..

[33] Darrow JJ, Sinha MS, Kesselheim AS (2018). When markets fail: patents and infectious disease products. Food Drug Law J..

[34] Warfel JM, Zimmerman LI, Merkel TJ (2014). Acellular pertussis vaccines protect against disease but fail to prevent infection and transmission in a nonhuman primate model. Proc. Natl Acad. Sci. USA.

[35] Jochems SP, Weiser JN, Malley R, Ferreira DM (2017). The immunological mechanisms that control pneumococcal carriage. PLoS. Pathog..

[36] Tacconelli E., Mazzaferri F., de Smet A.M., Bragantini D., Eggimann P., Huttner B.D., Kuijper E.J., Lucet J.-C., Mutters N.T., Sanguinetti M., Schwaber M.J., Souli M., Torre-Cisneros J., Price J.R., Rodríguez-Baño J. (2019). ESCMID-EUCIC clinical guidelines on decolonization of multidrug-resistant Gram-negative bacteria carriers. Clinical Microbiology and Infection.

[37] Fleming TR, Powers JH (2012). Biomarkers and surrogate endpoints in clinical trials. Stat. Med..

[38] Committee for Medicinal Products for Human Use (CHMP). Addendum to the guideline on the evaluation of medicinal products indicated for treatment of bacterial infections. European Medicines Agency, EMA/CHMP/351889/2013. http://www.ema.europa.eu/docs/en_GB/document_library/Scientific_guideline/2013/11/WC500153953.pdf (2013). Downloaded 1 May 2015.

[39] Miller FG, Joffe S (2011). Equipoise and the dilemma of randomized clinical trials. N. Engl. J. Med..

[40] Garattini S, Bertele V (2007). Non-inferiority trials are unethical because they disregard patients’ interests. Lancet.

[41] Graber MA, Ely J, Faine BA (2018). Are noninferiority studies ethically inferior?. Am. J. Health Syst. Pharm..

[42] Hey SP, Kesselheim AS (2017). Reprioritizing research activity for the post-antibiotic era: ethical, legal, and social considerations. Hastings Cent. Rep..

[43] Megiddo Itamar, Drabik Dusan, Bedford Tim, Morton Alec, Wesseler Justus, Laxminarayan Ramanan (2019). Investing in antibiotics to alleviate future catastrophic outcomes: What is the value of having an effective antibiotic to mitigate pandemic influenza?. Health Economics.

[44] Emanuel EJ, Wendler D, Killen J, Grady C (2004). What makes clinical research in developing countries ethical? The benchmarks of ethical research. J. Infect. Dis..

[45] Code of Federal Regulations. 45 C.F.R. § 46.111(a)(2) (“Criteria for IRB approval of research”), https://www.ecfr.gov/cgi-bin/text-idx?SID=855b4516c632063a1db33520391a59e8&mc=true&tpl=/ecfrbrowse/Title45/45cfr46_main_02.tpl (2018).

[46] Code of Federal Regulations. 21 C.F.R. § 56.111(a)(2) (“Criteria for IRB approval of research”), https://www.accessdata.fda.gov/scripts/cdrh/cfdocs/cfcfr/cfrsearch.cfm?fr=56.111 (2018).

[47] Rid A, Shah SK (2017). Substantiating the social value requirement for research: an introduction. Bioethics.

[48] Miller FG, Joffe S (2009). Limits to research risks. J. Med. Ethics.

[49] Weijer C, Miller PB (2004). When are research risks reasonable in relation to anticipated benefits?. Nat. Med..

[50] Doshi P (2017). Informed consent to study purpose in randomized clinical trials of antibiotics, 1991 through 2011. JAMA Intern. Med..

[51] Menikoff J (2017). What should patients be told about noninferiority studies?. JAMA Intern. Med..

[52] Joffe S, Miller FG (2012). Equipoise: asking the right questions for clinical trial design. Nat. Rev. Clin. Oncol..

[53] Gelinas L, Lynch HF, Bierer BE, Cohen IG (2017). When clinical trials compete: prioritising study recruitment. J. Med. Ethics.

[54] Anomaly, J. & Savulescu, J. Compensation for cures: why we should pay a premium for participation in ‘challenge studies’. *Bioethics.*10.1111/bioe.12596 (2019). 10.1111/bioe.12596 PMC6773221 31135070

[55] Temple R, Ellenberg SS (2000). Placebo-controlled trials and active-control trials in the evaluation of new treatments. Part 1: ethical and scientific issues. Ann. Intern. Med..

[56] Ellenberg SS, Temple R (2000). Placebo-controlled trials and active-control trials in the evaluation of new treatments. Part 2: practical issues and specific cases. Ann. Intern. Med..

[57] Powers JH, Fleming TR (2013). Noninferiority trials: clinical understandings and misunderstandings. Clin. Investig..

[58] Kaul S, Diamond GA (2006). Good enough: a primer on the analysis and interpretation of noninferiority trials. Ann. Intern. Med..

[59] Powers JH, Evans SR, Kesselheim AS (2018). Studying new antibiotics for multidrug resistant infections: are today’s patients paying for unproved future benefits?. Br. Med. J..

[60] Craig WA (1998). Pharmacokinetic/pharmacodynamic parameters: Rationale for antibacterial dosing of mice and men. Clin. Infect. Dis..

[61] Craig W (1993). Pharmacodynamics of antimicrobial agents as a basis for determining dosage regimens. Eur. J. Clin. Microbiol. Infect. Dis..

[62] Ambrose PG (2007). Pharmacokinetics-pharmacodynamics of antimicrobial therapy: It’s not just for mice anymore. Clin. Infect. Dis..

[63] Ambrose PG, Bhavnani SM, Ellis-Grosse EJ, Drusano GL (2010). Pharmacokinetic-pharmacodynamic considerations in the design of hospital-acquired or ventilator-associated bacterial pneumonia studies: look before you leap!. Clin. Infect. Dis..

[64] Ambrose PG (2008). Use of pharmacokinetics and pharmacodynamics in a failure analysis of community-acquired pneumonia: implications for future clinical trial study design. Clin. Infect. Dis..

[65] Peck CC, Rubin DB, Sheiner LB (2003). Hypothesis: a single clinical trial plus causal evidence of effectiveness is sufficient for drug approval. Clin. Pharmacol. Ther..

[66] Tyers, M. & Wright, G. D. Drug combinations: a strategy to extend the life of antibiotics in the 21st century. *Nat. Rev. Microbiol.* **17**, 141–145 (2019). 10.1038/s41579-018-0141-x 30683887

